# Bacterial Spectrum and Antibiotic Resistance Patterns of Ocular Infection: Differences between External and Intraocular Diseases

**DOI:** 10.1155/2015/813979

**Published:** 2015-10-21

**Authors:** Nan Wang, Qian Yang, Yiwei Tan, Liping Lin, Qiang Huang, Kaili Wu

**Affiliations:** Zhongshan Ophthalmic Center, State Key Laboratory of Ophthalmology, Sun Yat-Sen University, Guangzhou 510060, China

## Abstract

This study aimed to compare the differences of microbial spectrum and antibiotic resistance patterns between external and intraocular bacterial infections in an eye hospital in South China. A total of 737 bacteria isolates from suspected ocular infections were included in this retrospective study covering the period 2010–2013. The organisms cultured from the ocular surface (cornea, conjunctiva) accounted for the majority of the isolates (82.77%, *n* = 610), followed by the intraocular (aqueous humor, vitreous fluid), which accounted for 17.23% (*n* = 127). The top three species accounting for the external ocular infections were* S. epidermidis* (35.25%),* P. aeruginosa* (8.03%), and *S. simulans *(4.43%). The top three species for the intraocular infections were *S. epidermidis* (14.96%), *S. hominis* (8.66%), and *B. subtilis *(7.87%). The bacteria from the external ocular surface were more sensitive to neomycin, while those from the intraocular specimens were more sensitive to levofloxacin (*P* < 0.01). Multidrug resistance was found in 89 bacteria (12.08%), including isolates from both external (13.28%) and intraocular samples (6.30%). The results of this study indicate that the bacteria spectrum of external and intraocular infections is variable in the setting. A high percentage of bacterial organisms were found to be primarily susceptible to neomycin for external infection and levofloxacin for intraocular infection.

## 1. Introduction

Ocular bacterial infections can cause a series of symptoms and signs, such as the formation of pus, conjunctival hyperemia, lid edema, and even visual impairment. The causative bacteria can come from the outside environment or from systemic infections transported by blood. The eyelid and conjunctiva have normal bacterial flora, of which disequilibrium facilitates external or intraocular infection [1, 2]. Bacteria of the normal microbiome can also cause infection, especially when they enter the aqueous humor or vitreous fluid.

There have been many reports on the bacterial profile and antibiotic susceptibility of ocular infections, with varying results between cases [3–6]. For example, an original report from Japan analyzed the culture-positive rate and the prevalence of drug resistance among microorganisms isolated from discharges, corneal and conjunctival tissues, vitreous fluid, or aqueous humor of patients with ocular infections over a 4-year period [4]. Consequently, the major pathogen strains were gram-positive (GP) bacteria (*Staphylococcus *spp. mainly), and the levofloxacin-resistant strains accounted for 32.8% [4]. Bharathi and colleagues from South India reported that, of 4,417 ocular samples (from infections of the eyelid, conjunctiva, lacrimal apparatus, cornea, intraocular tissues, orbit, and sclera), the culture-positive rate of bacteria was 58.8%, with GP cocci being the most frequent bacteria isolated from ocular infections, which were sensitive to moxifloxacin (98.7%) and vancomycin (97.9%), while gram-negative (GN) isolates were more sensitive to amikacin (93.5%) and gatifloxacin (92.7%) [5]. In the New York Eye and Ear Infirmary, Adebayo and colleagues reviewed 12 years of data from their hospital and found that, among 20,180 conjunctival bacterial cultures, 60.1% were culture-positive and* S. aureus* was the most common pathogen; in addition, moxifloxacin and gatifloxacin currently appear to be the best choice for empirical broad-spectrum coverage [7]. Additionally, other reports on the ocular bacterial infection and antibiotic susceptibility have been reported in the literature from various countries, like Colombia [8], Pakistan [9], Italy [10], and Uganda [11], among other locations.

The intraocular infection, for example, bacterial endophthalmitis, is a common and serious condition that frequently leads to visual impairment and can even cause blindness [12]. A number of studies have demonstrated that the bacterial profile of endophthalmitis is different from the causative pathogens of ocular surface infection. Benz and colleagues from Miami reported that the most common organism identified in the vitreous fluid of endophthalmitis patients was* S. epidermidis* (27.8%) [13], while a report from London showed that the most common organism in the endogenous bacterial endophthalmitis is* S. aureus* [14]. In addition to the reports from various locations, limited data from the same hospital also clearly showed that the bacterial flora that infected the ocular surface was different from the pathogen bacteria of endophthalmitis [5, 6].

These different results, including the change of bacterial spectrum, have been attributed to the region and environment, as well as seasonal changes [15, 16]. To better understand the differences of bacterial profiles and resistance patterns between external and intraocular infections in South China, the present study retrospectively investigated and analyzed ocular isolates obtained from patients with suspected ocular infections. Additionally, the in vitro susceptibility of bacterial isolates from different ocular sites to eight antibiotics was assessed to provide guidance for clinical treatment.

## 2. Materials and Methods

A retrospective review was conducted on patients (outpatients and inpatients) who were suspected of having ocular infections based on their clinical findings and had undergone further microbiological evaluation at Zhongshan Ophthalmic Center, Guangzhou (northern latitude 23°6′′, eastern longitude 113°15′′), between January 2010 and December 2013, while patients either suspected or having a positive culture of viral, fungal or* Acanthamoeba* infection were excluded. This study was performed in compliance with the principles of the Declaration of Helsinki and was approved by the Institutional Ethics Committee of Zhongshan Ophthalmic Center, Sun Yat-Sen University.

### 2.1. Bacterial Isolation and Identification

Samples were taken from diseased tissues (i.e., cornea, conjunctiva, aqueous humor, and vitreous fluid) from all patients with suspected ocular infections (for ocular surface sampling, topical anesthesia by 0.5% proparacaine hydrochloride). All protocols were conducted as previously reported [17, 18]. Briefly, specimens of conjunctival sacs were collected by sterile cotton swabs; cornea specimens were sampled by scraping the base and edges of the ulcerated part of the cornea with a sterile special knife; anterior chamber fluids were aspirated through the limbus using a needle on a 1 mL syringe and vitreous specimens were obtained through the pars plana prior to antibiotic injection or vitrectomy. And then the collected samples were inoculated in nutrient broth overnight at 37°C. Subsequently, the broth was inoculated onto sheep blood agar for bacterial culture. The cultures were considered to be positive as Bourcier et al. reported [19], and bacteria isolates were identified using an automated microbiology system (Vitek 2 Compact, BioMerieux, Inc., Durham, NC, USA).

### 2.2. Antibiotic Susceptibility Test

Antibiotic susceptibility testing of isolated bacteria was performed in vitro on ceftazidime (30 *μ*g), cefuroxime (30 *μ*g), cefazolin (30 *μ*g), levofloxacin (5 *μ*g), ofloxacin (5 *μ*g), neomycin (30 *μ*g), tobramycin (10 *μ*g), and chloramphenicol (30 *μ*g) using the Kirby-Bauer disc diffusion method. Bacterial susceptibilities were recorded as “resistant,” “intermediate,” and “sensitive.” The antibiotic susceptibility was determined in accordance with the methods of the Clinical and Laboratory Standards Institute (CLSI). For the purpose of the study, “intermediate” and “sensitive” were both considered sensitive.

### 2.3. Statistical Analysis

The statistical analysis was performed using SPSS 17.0 (Chicago, IL, USA). The Chi-square test was employed for the comparison of categorical variables. Differences were considered to be significant at *P* < 0.05.

## 3. Results

A total of 3,040 samples from the suspected external and intraocular infections were cultured at our institution during the study period. Among the collected samples, 737 had culture-positive bacteria (Table 1). Of these isolates, the organisms cultured from the ocular surface (cornea, conjunctiva) accounted for the majority (82.77%, *n* = 610), followed by the intraocular (aqueous humor, vitreous fluid), which accounted for 17.23% (*n* = 127). The culture-positive rates for intraocular infections and the ocular surface were 19.21% (127/661) and 25.64% (610/2379), respectively. When the culture-positive rates of four types of samples were assessed, the highest rate was found in conjunctiva (48.85%; 213 of 436), followed by vitreous fluid (21.80%; 92 of 422), cornea (20.43%; 397 of 1943), and aqueous humor (14.64%; 35 of 239). The GP bacteria were the most prominent pathogen for external and intraocular infections (78.36% and 69.29%, resp.). The top three species for the external ocular infections were* S. epidermidis* (35.25%, *n* = 215),* P. aeruginosa* (8.03%, *n* = 49), and* S. simulans* (4.43%, *n* = 27). Specifically, the main causative organisms for cornea and conjunctiva infections were all* Staphylococcus* spp. The top three species for the intraocular infections were* S. epidermidis* (14.96%, *n* = 19),* S. hominis* (8.66%, *n* = 11), and* B. subtilis* (7.87%, *n* = 10).

**Figure 1:**
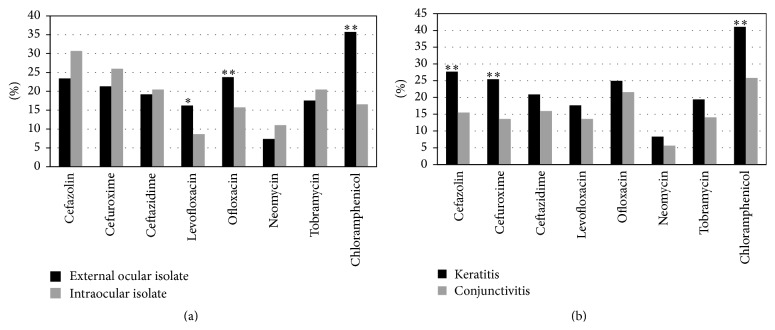
The drug-resistance of bacteria isolates from different ocular tissues to eight antibiotics. (a) Comparison of external ocular (black) and intraocular (gray) bacteria isolates; (b) comparison of cornea (black) and conjunctiva (gray) isolates. ^*∗*^ *P* < 0.05, ^*∗∗*^ *P* < 0.01.

A comparison of the antibiotic resistance of external and intraocular bacteria to ceftazidime, cefuroxime, cefazolin, levofloxacin, ofloxacin, neomycin, tobramycin, and chloramphenicol was shown in Figure 1(a). Generally, among five antibiotics that are present in eye drop products in China, the bacteria found in the external ocular were more sensitive to neomycin, while bacteria from intraocular isolates were significantly more sensitive to levofloxacin than to neomycin (*P* < 0.01). Meanwhile, for the cephalosporins, the intraocular isolates showed a high sensitivity to ceftazidime. There were significant differences in resistance to levofloxacin and chloramphenicol between external and intraocular isolates (*P* < 0.05). The antibiotic resistance of* S. epidermidis* in both external and intraocular infections showed no significant differences compared to these eight antibiotics. The comparison of the antibiotic resistance of cornea and conjunctiva bacteria isolates to eight antibiotics was shown in Figure 1(b). On the whole, cornea bacteria isolates exhibited significantly higher resistance to cefazolin, cefuroxime, and chloramphenicol as compared to conjunctival isolates (*P* < 0.01).

Additionally, multidrug resistance (MDR) bacterial species were found. We found 89 (12.08%) MDR bacteria; those were resistant to at least one agent in each of three or more antibacterial categories (in our study, cephalosporins, quinolones, aminoglycosides, and phenicols) (Table 2). Of these, the percentage of MDR strains from external and intraocular isolates was 13.28% (*n* = 81) and 6.30% (*n* = 8), respectively. The MDR strains of* P. aeruginosa*,* K. roseus*,* E. cloacae*, and* Stenotrophomonas maltophilia* were found in both the external and intraocular isolates. However, there were no MDR strains of* Staphylococcus *spp. in the intraocular infections.  Table 3 displayed the raw numbers of MDR strains that were resistant to eight antibiotics. The highest resistance rate of MDR strains was seen for chloramphenicol (86.53%, 77/89), including 88.52% (54/61) in keratitis, 85% (17/20) in conjunctivitis, and 75% (6/8) in endophthalmitis.

## 4. Discussion

The identification of causative pathogens and antibiotic susceptibility tests are important in clinical practice. Our study revealed that the major causative pathogens for both external and intraocular infections were* Staphylococcus *spp., specifically 61.64% for external and 43.31% for intraocular infection. Furthermore, we also found that* P. aeruginosa*, the second most common pathogen for ocular surface (8.03%), was less frequently detected in the aqueous humor (5.71%) and vitreous fluid (0.24%). Studies conducted by Bharathi et al. in India reported that the most common bacterial species were different among infections of eyelid, conjunctiva, cornea, lacrimal apparatus, and intraocular tissue, of which* S. pneumoniae* (35.9%) were the predominant bacteria of infective keratitis in India [5, 20, 21]. In Northeastern United States, Chen and Adelman reported that, among 143 culture-positive isolates, the most prevalent bacteria causing endophthalmitis were* coagulase-negative Staphylococcus* (37.5%),* Viridans Streptococcus* (11.3%), and* Streptococcus pneumoniae* (6.9%) [16]. The difference between their results and those presented in the present study may be due to the variation of geographic location, climate, or contact lens [20, 22]. Besides, compared to Bharathi et al.'s study with 58.8% culture-positive bacteria in 4417 ocular samples [5], we found a lower total culture-positivity rate (24.24%); similar prevalence within China has been reported from Beijing (28.6%, 1339/4705) [23], Zhejiang (15.8%, 138/871) [24], and Tianjin (34.3%, 1119/3265) [25], which may be attributable to the use of antibiotics, obtained from a local physician or a drug store, before patient came to our hospital, or overdiagnose cases of “red eye” as bacterial conjunctivitis and keratitis.

In our study, 8 antibiotics belonging to four categories (cephalosporins, quinolones, aminoglycosides, and phenicols) were tested for resistance. The antibiotic susceptibility analysis showed variation between external and intraocular isolates: the bacteria from the ocular surface were more sensitive to neomycin, while the intraocular isolates presented higher sensitivity to levofloxacin. These results are different from those of a study from Bangalore, in which Hemavathi and colleagues reported that the organisms from both external and intraocular isolates were susceptible to quinolone antibiotics [6]. Considering that eye drops with quinolones (levofloxacin, ofloxacin) and aminoglycosides (neomycin, tobramycin) are the major eye drop products in the Chinese market, the present data will aid in choosing eye drops for ocular surface infections. Though eye drops containing ceftazidime are not commercially available because of its rapid degradation in aqueous solutions, for serious intraocular infections such as suppurative endophthalmitis, systemic or intraocular application of antibiotics (e.g., ceftazidime) is necessary. Studies showed that recent trends have shifted to using ceftazidime, instead of aminoglycosides that have toxic role in macula, to treat endophthalmitis infected with gram-negative bacteria [12, 26, 27]. Our current result showed that intraocular isolates had a high sensitivity to ceftazidime, which could be a better choice for intraocular application. Additionally, studies on the optimization of liposomal encapsulation for developing a potential eye drop formulation of ceftazidime have been reported [28, 29], which may provide more effective treatment for ocular infections. No matter external and intraocular infections, our present data will help clinician make decisions for the choice of antibiotics before definitive information on the causative pathogenic microorganisms is available. MDR organisms have been recently defined as those that are resistant to at least one agent in each of three or more antibacterial categories, introduced by the European Centre for Disease Prevention and Control in 2012 [30]. Following this new definition, the total rate of MDR strains was 12.08% in our study, wherein the rate of MDR* S. epidermidis* and* P. aeruginosa* accounted for approximately 22.47% and 11.24%, respectively. By defining MDR to two or more drugs, Muluye et al. reported that 87.1% of isolates showed MDR among 62 bacteria isolated from eye discharge samples [31]. Although it is difficult to compare our MDR results with other reports, MDR bacteria have been shown to be very important in ocular infections in previous studies [31–33]. It has been suggested that the indiscriminate, prolonged use of a wide range of antibiotics may be a major factor in the development of drug resistance [34]. The combined use of antibiotics could provide broader coverage against infection prior to susceptibility testing.

In conclusion, we found that the bacteria profile of external and intraocular infections varied in the setting of our study. In the comparison of eight antibiotics, the bacteria from external ocular samples were more sensitive to neomycin, while intraocular isolates were significantly more sensitive to levofloxacin than to neomycin. A higher MDR appeared in ocular surface isolates. It should be noted that there were still some limitations in our study, such as the limited kinds of antibiotics, the relatively lower intraocular species, and the fact that the results of culture-positive and antibiotic susceptibility in vitro do not always agree with clinical observations. The in vitro results are dependent on the protocol of sample collection and inoculation, as well as primary treatment before sample collection.

## Figures and Tables

**Table 1:** Bacterial isolates recovered from external and intraocular infections.

	External ocular isolates		Intraocular isolates		Total	
	Number (cornea, conjunctiva)	%	Number (aqueous humor, vitreous)	%	Number	%
Gram-positive organisms^*∗*^	**478 (294, 184)**	**78.36**	**88 (24, 64)**	**69.29**	**566**	**76.8**
*Staphylococcus *spp.	376 (223, 153)	61.64	55 (15, 40)	43.31	431	58.48
*Kocuria *spp.	27 (21, 6)	4.43	4 (1, 3)	3.15	31	4.21
*Micrococcus *spp.	5 (5, 0)	0.82	0	0.00	5	0.68
*Bacillus *spp.	8 (5, 3)	1.31	10 (3, 7)	7.87	18	2.44
*Enterococcus *spp.	5 (4, 1)	0.82	1 (0, 1)	0.79	6	0.81
*Corynebacterium *spp.	4 (4, 0)	0.66	0	0.00	4	0.54
*Streptococcus *spp.	6 (3, 3)	0.98	8 (3, 5)	6.30	14	1.9
*Aerococcus *spp.	5 (3, 2)	0.82	0	0.00	5	0.68
Others	42 (26, 16)	6.89	10 (2, 8)	7.87	52	7.06
Gram-negative organisms	**132 (103, 29)**	**21.64**	**39 (11, 28)**	**30.71**	**171**	**23.2**
*Pseudomonas *spp.	56 (51, 5)	9.18	5 (2, 3)	3.94	61	8.28
*Burkholderia *spp.	10 (8, 2)	1.64	0	0.00	10	1.36
*Acinetobacter *spp.	10 (6, 4)	1.64	1 (0, 1)	0.79	11	1.49
*Escherichia *spp.	6 (5, 1)	0.98	3 (2, 1)	2.36	9	1.22
*Enterobacter *spp.	6 (5, 1)	0.98	7 (2, 5)	5.51	13	1.76
*Serratia *spp.	5 (4, 1)	0.82	0	0.00	**5**	0.68
*Chryseobacterium *spp.	3 (3, 0)	0.49	0	0.00	3	0.41
*Gardnrella vaginallis*	1 (0, 1)	0.16	0	0.00	1	0.14
*Neisseria *spp.	1 (0, 1)	0.16	0	0.00	1	0.14
*Xanthomonas *spp.	2 (0, 2)	0.33	3 (0, 3)	2.36	5	0.68
*Sphingomonas *spp.	0	0.00	3 (0, 3)	2.36	3	0.41
*Shigella *spp.	0	0.00	2 (1, 1)	1.57	2	0.27
Others	32 (21, 11)	5.25	15 (4, 11)	11.81	47	6.38
Total	**610 (397, 213)**	**100.00**	**127 (35, 92)**	**100.00**	**737**	**100**

^*∗*^ *P* < 0.05: gram-positive isolates between external and intraocular infections (95% CI: 0.41~0.95).

**Table 2:** Comparison of MDR bacteria between external and intraocular infections.

	External ocular infection		Intraocular infection	
	Cornea	Conjunctiva	Aqueous humor	Vitreous fluid
*S. epidermidis*	13 (119)	7 (96)	0 (5)	0 (14)
*P. aeruginosa*	9 (45)	0 (4)	1 (2)	0 (1)
*B. cepacia *	6 (8)	1 (2)	0 (0)	0 (0)
*S. hominis*	4 (19)	1 (8)	0 (5)	0 (6)
*E. coli*	3 (5)	0 (1)	0 (2)	0 (1)
*S. auricularis*	3 (14)	0 (5)	0 (1)	0 (2)
*K. roseus*	3 (11)	0 (3)	0 (0)	2 (2)
*S. simulans*	3 (17)	0 (10)	0 (1)	0 (1)
*S. haemolyticus*	3 (15)	2 (5)	0 (0)	0 (6)
*S. warneri*	3 (14)	0 (6)	0 (0)	0 (3)
*E. faecalis*	2 (2)	1 (1)	0 (0)	0 (1)
*A. junii*	2 (2)	0 (2)	0 (0)	0 (1)
*A. baumannii *	1 (2)	0 (1)	0 (0)	0 (0)
*K. varians*	1 (3)	0 (0)	0 (0)	0 (0)
*P. putida*	1 (2)	0 (0)	0 (0)	0 (0)
*Methylobacterium*	1 (2)	0 (0)	0 (0)	0 (0)
*K. kristinae*	1 (7)	1 (3)	0 (1)	0 (1)
*Moraxella lacunata*	0 (3)	1 (4)	0 (0)	0 (0)
*P. stutzeri*	1 (2)	0 (1)	0 (0)	0 (2)
*E. cloacae*	1 (3)	0 (0)	0 (2)	2 (4)
*Stenotrophomonas maltophilia*	0 (0)	1 (2)	0 (0)	1 (3)
*S. thoraltensis*	0 (0)	0 (0)	0 (0)	1 (1)
*Vibrio alginolyticus*	0 (0)	0 (0)	0 (0)	1 (1)
*S. lentus*	0 (6)	4 (10)	0 (1)	0 (2)
*N. gonorrhoeae*	0 (0)	1 (1)	0 (0)	0 (0)
Total number	**61**	**20**	**1**	**7**
Percentage (%)^*∗*^	**13.28**		**6.3**	

^*∗*^ *P* < 0.05 external versus intraocular infection (95% CI: 0.20~0.91).

**Table 3:** The numbers of MDR strains resistant to eight antibiotics.

	Cefazolin	Cefuroxime	Ceftazidime	Levofloxacin	Ofloxacin	Neomycin	Tobramycin	Chloramphenicol
Keratitis^*∗*^	33 (10, 23)	33 (11, 22)	28 (22, 6)	35 (22, 13)	46 (31, 15)	22 (13, 9)	40 (23, 17)	54 (30, 24)
Conjunctivitis	7 (5, 2)	8 (7, 1)	13 (12, 1)	9 (7, 2)	13 (11, 2)	6 (6, 0)	13 (10, 3)	17 (14, 3)
Endophthalmitis	6 (2, 4)	5 (1, 4)	5 (3, 2)	3 (2, 1)	5 (3, 2)	3 (1, 2)	6 (3, 3)	6 (1, 5)
Total	46 (17, 29)	46 (19, 27)	46 (37, 9)	47 (31, 16)	64 (45, 19)	31 (20, 11)	59 (36, 23)	77 (45, 32)

^*∗*^MDR number (gram-positive, gram-negative strains).
